# Rural-urban variation in incidence of psychosis in France: a prospective epidemiologic study in two contrasted catchment areas

**DOI:** 10.1186/1471-244X-14-78

**Published:** 2014-03-17

**Authors:** Andrei Szöke, Thomas Charpeaud, Anne-Marie Galliot, Jeanne Vilain, Jean-Romain Richard, Marion Leboyer, Pierre-Michel Llorca, Franck Schürhoff

**Affiliations:** 1 AP-HP, Groupe Hospitalier “Mondor” Pôle de Psychiatrie, Créteil 94000, France; 2 INSERM, U955 Equipe 15, Créteil 94000, France; 3 Université Paris-Est, Faculté de médecine, Créteil 94000, France; 4 Fondation Fondamental, Créteil 94000, France; 5 Université d’Auvergne, Clermont-Ferrand 63000, France; 6 CHU Clermont-Ferrand, Clermont-Ferrand 63000, France

## Abstract

**Background:**

The aim of our study is to provide data on the incidence of psychotic disorders in France and compare the incidence rates in populations with different levels of urbanization.

**Methods:**

We prospectively included the incident cases of psychotic disorders from two catchment areas with contrasted levels of urbanization. In the more rural area, we also calculated incidence rates in three different groups of population defined by the size of towns in which they live (small, medium and large towns).

**Results:**

The annual incidence of psychosis was greater in the urban area (36.02/100000 person-year at risk) than in the rural area (17.2/100000 person-year at risk).

Non-affective psychoses were the majority of cases and their incidence was greater in males and younger subjects. The affective psychoses were slightly more frequent in women and showed less variation with age. In the rural centre, greater levels of urbanicity were associated with an increase in the incidence of all psychoses (affective and non-affective).

**Conclusions:**

Our study confirms previous observations of increased incidence rates for non-affective psychoses in the more urbanized areas and suggests that a similar pattern might be present for affective psychoses.

## Background

Psychotic symptoms, defined as either hallucinations or delusions, constitute the essential features of psychotic disorders but may also be seen in other psychiatric conditions. Despite the classical Kraepelinian dichotomy between psychotic and affective disorders, data have consistently shown that these disorders share at least some aetiological factors. In particular, it has been hypothesized that the origin of psychotic symptoms is similar across different diagnostic categories and thus between affective and non-affective psychosis [1].

Studies of incidence rates of these disorders and their variation are a crucial step in unravelling the aetiology of this group of disorders [2]. Such studies are also important for measuring the burden associated with these disorders and inform public health policies on most efficient spatial distribution of mental health resources [3].

Several studies and reviews have analysed the incidence of schizophrenia in different settings and the factors influencing it [2]. The incidence of schizophrenia shows important geographical variation across countries, between rural and urban sites and even at a single city level, between neighbourhoods [4].

Despite differences in the definition of urbanicity (place of birth, of upbringing or of residence) and method to measure it (town size, population density), an excess of incident cases of schizophrenia [5] and non-affective psychosis [3] in the most urbanized environments emerged as a robust finding. Moreover, there is evidence for a dose–response relationship between urbanicity and risk for schizophrenia e.g. [6,7]. These findings are of concern given that most of the World’s population already lives in urban areas and the proportion of people living in cities will continue to increase.

Fewer studies of geographical variation in incidence of affective psychoses between rural–urban areas or at smaller scale (i.e. neighbourhoods of a city) have been published [5,8-10]. As a whole, those studies suggest that affective psychoses show less spatial variation but, to date, evidence is too limited to draw definite conclusions.

Another limitation of the available data on geographical variation of psychosis incidence is that the majority of the studies took place in a limited number of countries (mainly the UK and countries from Northern Europe). Similar studies, in different contexts, are needed to confirm and expand these findings and test hypotheses generated by previous research.

The present study is part of a larger effort designed to explore gene-environment interactions in the aetiology of psychosis (EUropean network of national schizophrenia networks studying Gene-Environment Interactions: http://www.eu-gei.eu). The environmental part of EU-GEI aims to measure the variation in the incidence of psychotic disorders and affective psychoses across five European countries (the United Kingdom, the Netherlands, Italy, Spain and France) and, in each country, between urban and rural areas. Analyses of putative risk factors, genetic and environmental, at individual and area level, that could influence the occurrence of the disorders and influence their incidence will complement this descriptive part.

This article presents results after 2 years of data collection on incidence of affective and non-affective psychosis in France, and compares incidence rates between populations with different levels of urbanisation.

## Methods

### Subjects

We included data from all subjects residing, at the time of their inclusion, in two catchment areas (see description below), that were between 18 and 64 years old and came in contact with a psychiatrist, for the first time, for a psychotic episode (diagnosis of psychotic disorder or mood disorders with psychotic features according to DSM IV). All subjects suffering from psychosis due to medical general conditions (i.e. when there was evidence that the delusions or hallucinations were the direct consequence of a general medical condition) or from substance-induced psychosis were excluded.

### Catchment areas and at-risk populations

The study has been conducted in two tightly defined catchment areas of similar population sizes. However, although one area, in the Paris region (Val de Marne department), is highly urbanized the other is a largely rural area in the centre of France (Puy de Dôme department).

To further explore the role of urbanization, we decided to group the population from the rural area, based on the size of towns, into three groups of approximately equal size. Thus, we defined a population group living in the smallest towns in the area (between 60 and 1350 inhabitants), one living in the medium towns (between 1350 and 4650 inhabitants) and finally a population group living in the largest towns in the area (between 4650 and 19124 inhabitants).

Table 1 summarizes the main characteristics of the catchment areas and population groups.

**Table 1:** Catchment areas and population groups

	**Urban**	**Rural**			
		**Total**	**Smallest towns**	**Medium towns**	**Largest towns**
Total population	209198	187516	62974	63201	61341
At Risk population (18–64 y)	133239	113534	37907	38812	36818
Population density/ km^2^	7790	70.6	NA*	NA*	NA*
Number of towns	7	164	130	27	7

*As the towns that constitute each of the three groups of population in the rural area are non-adjacent the population density is not available (NA).

### Organization of the study

Before the beginning of the study we contacted all psychiatrists, with public or private practice, that work in the two areas and asked them to participate in the study. All public services (emergency wards, in- and out- patient clinics) and private clinics were involved in the study.

At the beginning of the study, and then at yearly intervals, we held meetings in order to present to all involved psychiatrists the procedures for the identification and reporting of cases and answer their questions. In addition, we provided clear written instructions to all participant psychiatrists, and they had the possibility to contact the researchers at any time if they had any questions.

For each facility employing several psychiatrists (hospital, outpatient clinic, etc.) we tried to directly involve one of them in the study, in order to supervise the identification and reporting of new cases. When this was not possible one researcher contacted the facility on a regular basis to remind the study methodology and inquire about new cases.

Data reported here encompass two years of data collection at each site: from June 2010 to May 2012 in the urban area and from September 2010 to August 2012 in the rural area.

### Data collection

The treating psychiatrist reported each new case using an anonymous, standard form. The form comprised inclusion/exclusion criteria and a list of symptoms that allowed the researchers to generate probable diagnosis. It also included basic demographics (gender, age) and the town of residence or, for larger towns, an area code that corresponds to around 3000 people (the “IRIS” code developed by the French National Institute for Statistics [INSEE] for reporting census data).

Patients received information about the study and, even as precautions had been taken to ensure anonymity, they had the opportunity, in accordance with ethics committee recommendations, to oppose the communication of their data. In this case, the physician addressed to the research team a blank form (to signal a new case).

To avoid counting a subject twice (for example if a subject presented him/herself to two different physicians and did not mention it), in the case of forms containing the same demographics and area code, only one form was kept.

To avoid differential incidence rates between centres arising from differences in methods used, we used identical procedures in the two centres. In addition, we held regular meetings involving researchers from the two centres to ensure convergence of methodology throughout the study period.

### Data reporting

We present raw incidence rates for non-affective psychoses (i.e. disorders under the heading of “schizophrenia and other psychotic disorders” in DSM IV), affective psychoses (“mood disorders with psychotic features” in DSM IV) and all psychoses (the sum of the two previous categories) detailed by centre, gender and age interval. The incidence rates measure the number of new cases observed for 100000 person-years at risk. Consistent with the DSM IV criteria, a non-affective disorder was recorded either in the absence of affective/mood symptoms or when delusions and/or hallucinations were present, in the absence of mood symptoms, for a period of at least two weeks. We chose the age bands to report our data (18–24 / 25–39 / 40–54 / 55–64) in accordance to the age bands used to report data from the French census.

In order to compare data between the two centres and, for the rural centre, between small, medium and larger towns, we standardized incidence rates according to age and gender using direct standardization methods [11]. We used the age and gender structure of the total population of mainland France for this purpose.

All data for the population denominator was extracted from the 2008 census, the latest available at the time of analysis.

### Ethical approval

The relevant Regional Ethical Committee (Comité de Protection des Personnes – CPP Ile de France IX) examined and approved the study protocol (project number 2010-A00161-38) in accordance with the Helsinki Declaration.

For this first, descriptive, part of the study written consent was not requested because the Ethical Committee agreed that, for ethical reasons, it was important to preserve anonymity of the subjects. Thus, all data sent to the researchers by the treating psychiatrists were anonymous and the patients were not in contact with the research team. However, the patients received from their treating psychiatrist written information about the study (approved by the ethical committee mentioned above) and had the opportunity to oppose the communication of their data.

## Results

### Raw incidence rates and variation according to gender and age

In the urban area, 96 cases of psychosis have been reported which corresponds to a raw incidence of 36.02/ 100000 person-years at risk. The mean age of the subjects was of 33.2 years and 55.3% were males (Table 2).

**Table 2:** Comparison of number of cases and raw incidence rates in urban and rural centres and rural towns of different sizes

	**Affective psychoses**		**Non-affective psychoses**		**All psychoses**	
	**Number of cases**	**Incidence**^**a **^**(raw)**	**Number of cases**	**Incidence**^**a **^**(raw)**	**Number of cases**	**Incidence**^**a **^**(raw)**
Urban area	38	14.26	56	21.76	94	36.40
Rural area (global)	13	5.73	26 ^b^	11.45	39	17.18
Rural area (biggest towns)	7	9.51	17	23.09	24	32.59
Rural area (medium towns)	4	5.15	6	7.73	10	12.88
Rural area (smallest towns)	2	2.64	1	1.32	3	3.96

^a^Per 100000 at risk subjects*year.

^b^Includes 2 homeless subjects.

In the rural area, 39 cases of psychosis have been reported which corresponds to a raw incidence of 17.2/ 100000 person-years. 71.8% were males and the mean age was of 34.4 years.

**Figure 1:**
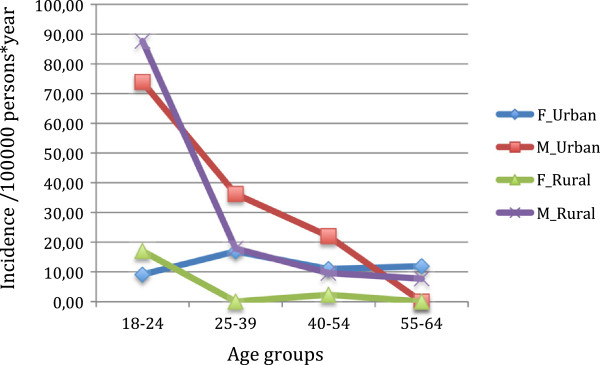
Observed incidence of non-affective psychoses by centre and gender.

As expected, non-affective psychoses were more frequent in males (in both areas) and in the youngest age band. Differences between genders and between age bands were more pronounced in the rural area. The incidence rate ratio (IRR) for gender (incidence rate in men compared with incidence rate in women) was of 7.68 in the rural area (CI = 6.73-8.88) and of 2.44 (CI = 1.88-3.00) in the urban area. The IRR based on the comparison of the incidence in the 18–24 age band with the incidence in the 55–64 band was of 14.05 (CI = 12.56-15.53) in the rural area and of 6.30 (CI = 5.08-7.53) in the urban area (Figure 1).

**Figure 2:**
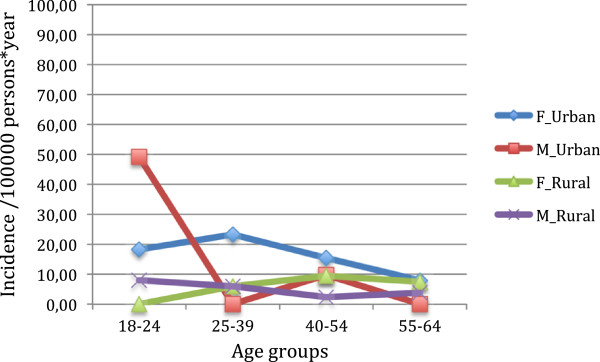
Observed incidence of affective psychoses by centre and gender.

Affective psychoses were slightly more frequent in females (24 vs. 14 in the urban area and 8 vs. 5 in the rural area). The age pattern was different between centres: small differences between age groups in the rural area and an excess of cases in the younger (male) subjects in the urban centre (Figure 2).

### Comparison between centres and, in the rural centre, between towns of different sizes

Compared with raw incidence rates, standardized incidence rates were slightly larger in the rural area and slightly smaller in the urban area (Table 2 and Table 3).

**Table 3:** Comparison of standardized incidence rates between urban and rural centres and rural towns of different sizes

	**Affective psychoses**		**Non-affective psychoses**		**All psychoses**	
**Annual incidence/100000**	**Incidence rate**	**IRR (CI)**	**Incidence rate**	**IRR (CI)**	**Incidence rate**	**IRR (CI)**
Urban area	13.76	2.39^a^ (1.27-4.49)	21.32	1.61^a^ (1.03-2.50)	35.08	1.86^a^ (1.29-2.66)
Rural area (global)	5.76	1	13.23	1	18.90	1
Rural area (biggest towns)	9.50	3.86^b^ (0.75-19.69)	24.50	11.09^b^ (2.23-55.03)	34.01	7.28^b^ (2.36-22,44)
Rural area (medium towns)	5.26	2.14^b^ (0.37-12.30)	9.45	4.28^b^ (0.78-23.37)	14.71	3.15^b^ (0.94-10.52)
Rural area (smallest towns)	2.46	1	2.21	1	4.67	1

^a^Incidence rate in the rural area (global) as reference.

^b^Incidence rate in the “smallest towns” as reference.

**Figure 3:**
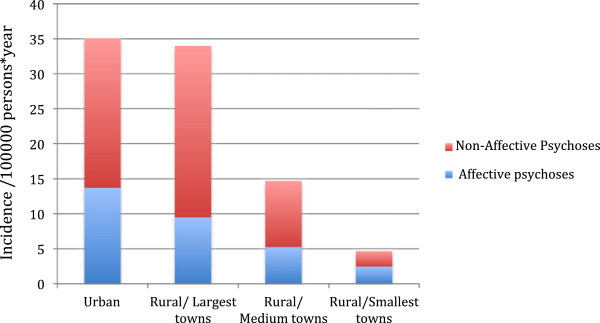
Incidence rates for affective and non-affective psychoses detailed by degree of urbanization.

As seen in Table 3, all incidence rates were elevated in the urban centre in comparison with the rural centre. When we divided the data from the rural centre according to the size of the towns (rough measure of the urbanization level), we observed the same trend of higher incidences in the more urbanized settings. Furthermore, incidence figures for the more urban zones were close to those observed in the urban centre (Figure 3).

## Discussion

In this article, we report the incidence rates of affective, non-affective and all psychoses in two tightly defined, contrasted catchment areas (rural vs. urban), in France. For both affective and non-affective psychoses, incidence rates were increased in the urban area and in the most urbanized populations of the rural area.

Our discussion will focus first on our methodological choices, strengths and potential limitations and how they could affect results. We then discuss our results in comparison with results from similar studies.

### Methodological choices, strengths and potential limitations

First of all, our results are based on incident cases from secondary/tertiary care. Given the severity of these disorders and the fact that in the two areas involved in our study psychiatric services, public and private, are easily accessible, it is probable that this represents a vast majority of the cases of incident psychoses. However, as we did not specifically address the issue of cases that are seen only in primary care, our results should not be generalized to all cases of psychosis.

Urbanicity is probably a proxy for some underlying, as yet unidentified, risk factor. Several hypotheses regarding the exact risk factors have been advanced [5]: biological, socio-economic or psychological (vitamin D insufficiency, elevated risk for infections, higher levels of everyday stresses, lower social capital and higher social fragmentation, etc.) but none has been validated to date. For this reason, there is debate on how best to define urbanicity and the period of risk associated with it [12]. For practical reasons, we chose to define urbanicity as a function of place of residence. It has been argued that place of birth (or even the place where the foetal period of development took place) has to be used rather than the place of upbringing or of residence. However, the influence of this choice on the results is limited by the fact that urban birth and urban residence are strongly associated [13].

The accuracy of the incidence rates depends on our capacity to identify all new cases of psychosis. Cases could be missed if patients are cared for outside the catchment area or if cases from the catchment area are not reported. In France, public psychiatric facilities offer care on a strictly catchment-area basis (“secteur psychiatrique”). The same restrictions do not apply to private practice and thus we cannot exclude that some patients are cared for outside the catchment areas. However the number of private psychiatrists in the two areas was substantial. Therefore, the probability that subjects with first psychotic episodes, who experience important behavioural, cognitive and volitional difficulties, travelled outside the area for psychiatric care seems small.

To ensure adequate reporting of new cases, we made every effort to involve a majority of the psychiatrists from the two catchment areas. Only a small proportion of psychiatrists, all with individual private practice, and all from the urban centre have declined participation to the study. This could result in an underestimation of the incidence rates. However, based on the small number of cases reported by the participating psychiatrists with individual private practice and the proportion of non-participating psychiatrists, we estimate that this could not significantly impact the reported rates. Furthermore, the impact is limited to the urban centre and thus could not affect our conclusions of greater incidence rates compared to the rural centre (or differences between populations with different levels of urbanization in the rural centre).

The procedures to identify and classify cases also deserve some comments. To identify new cases, we used a prospective, standardized methodology involving the reporting of symptoms present and not of specific diagnosis. The anonymous reporting of the cases helped to limit the proportion of patients that opposed the communication of their data. However, this also meant some inherent limitations as the number of details on each case has to be limited (to avoid indirect identification) and a test of inter-rater reliability could not been conducted.

We adopted this method to simplify reporting (and thus limit the risk of not reporting cases), enhance reliability and avoid potential problems with different diagnostic procedures used by different psychiatrists.

We decided to classify cases as affective or non-affective psychosis for several reasons. Firstly, this classification is based on a restricted number of symptoms (hallucinations, delusions, depression or elated mood) and avoids symptoms that tend to show low inter-rater agreement (e.g. formal thought disorder, affect flattening etc.) e.g. [14,15]. Second, longitudinal studies (e.g. [16]) showed that first episode diagnosis could change over time in a sizable proportion of cases. However, a majority of these changes are between different diagnoses of non-affective psychoses (e.g. from brief psychotic disorder to schizophrenia) or between diagnoses of affective psychoses (e.g. from unipolar to bipolar psychosis). Furthermore, the usually short time of observation until cases have been reported would inherently lead to diagnostic uncertainties and potential misclassification had a more specific diagnostic been used (e.g. observation before reporting the case was usually less than the 6 month interval required for a definite schizophrenia diagnosis).

Summing up all these arguments, we are confident that even if an underestimation of cases cannot be ruled out, it would not significantly influence the incidence rates and, more importantly, would not bias the results between centres. A more accurate estimation of potentially missed cases could be achieved by a leakage study. Such a study is planned at end of the data collection period of the EU-GEI study, which is scheduled for mid-2014.

### Comparison of our results with data from the literature

Comparing our data with previous data from France seems difficult. To our knowledge, the last published data are more than 30 years old [17]. In the cited article, for the 1973–1982 period, first-time hospitalizations for schizophrenia were, at national level, around 10/100000 at risk population. Several important methodological differences with the present study (diagnostic criteria used, categories of diagnoses reported, population at risk, etc.) limit the interest of this comparison.

In sharp contrast with the lack of epidemiological data in France, a recent review [3] found more than 80 reports on incidence of psychosis in England between 1950 and 2009.

Compared with data from this review, our data show several similarities: affective psychoses show lower incidences relative to non-affective psychoses, non-affective psychoses rates are elevated in men compared to women and more so before mid-life. For affective psychoses, in the cited review, there are less gender differences in incidence rates (when they exist, they are, as in our study, in the direction of greater incidence in women). The only notable difference is that our data did not show a clear peak in the twenties and second peak in the late forties for non-affective psychoses in women. More research is needed to confirm or infirm this difference in pattern.

In the cited review of studies of incidence of psychosis in England [3] there is no study that assessed the incidence of affective and non-affective psychoses in both a rural and an urban setting. Thus, we chose for comparison data from two recent studies which showed the greatest contrast in terms of urbanicity: one from East London [18] and one from Northumberland [19]. With the exception of affective psychoses in the urban centre which rate is similar to ours (13.5), rates in England are higher than those we observed in France (non-affective psychoses urban 36.8, rural 17.8 and affective psychoses in the rural area 8.6) but there is a similar general pattern of more non-affective than affective psychoses and greater incidence rates in the urban as opposed to the rural site.

Studies that concomitantly explored incidence of psychoses, using the same categories as we did (i.e. affective, non-affective and all psychoses), in both rural and urban settings are more useful as a comparison for the pattern of urban–rural differences observed in our study. To our knowledge, there are only 5 such studies in the literature. Four of them, originating from north European countries, are based on national psychiatric registries and compare rates of non-affective psychoses [20,21] or both non-affective and affective psychoses [8,9] according to the degree of urbanization. With the exception of the study by Marcelis et al., that included subjects between 14 and 50 years old, the other studies had a restricted range of age for at risk population (16 to 25 for one study, over 25 for another, less than 22 for the third) thus preventing any generalization of their results. However, despite this limitation and different definitions of urban/rural exposures (either at birth or at the time of diagnosis) and levels of urbanicity the four studies suggest that incidence of non-affective psychoses (including schizophrenia) is increased by the degree of urbanization. In the Eaton et al. study [9], for affective psychoses (including bipolar psychoses), there was not a clear trend (both extremes of urbanicity showing a small excess of cases and the middle category showing the smallest incidence rate) but this study was restricted only to cases with a first diagnosis before age 22. In the Marcelis et al., study [8] affective psychoses showed a similar trend to non-affective psychoses (higher risk in more urban areas) but with a lower relative risk.

Because it is similar to our study in many methodological aspects, a recent study from Ireland [5] deserves more comments. The authors used a similar design, prospectively collecting data on subjects at their first contact with psychiatric services for psychotic symptoms, in two catchment areas: one urban and one rural. A major difference from our study is that they did not use exclusion criteria based on age or aetiology (due to medical general conditions or use of toxic substances).

The results from this study are detailed by diagnostic category, gender and catchment area (urban vs. rural). They are at odds with our results and most of the results from the literature as they show, as a whole, larger incidence rates for psychoses (with the exception of schizophrenia) in the rural areas. These differences (in affective, non-affective and overall psychoses) are essentially driven by very high incidence rates in females in the rural area (36.7 per 100000). Almost half of the total cases of psychoses in this category of subjects (i.e. 18.3 per 100000) are non-affective psychoses other than schizophrenia raising the question about the contribution of secondary psychoses (due to medical conditions, to dementia or substance induced) to the data.

## Conclusions

Our findings on non-affective psychoses are consistent with previous reported data on the influence of age, gender and urbanicity with the possible exception of incidence rate variation according to age in women. Our results suggest that, even in a globally rural area, incidence is still influenced by the size of a city.

For affective psychoses, we found a slight excess of incidence in women and a lesser influence of age. We also found results similar to those of non-affective psychoses when incidence rates were compared according to urbanicity i.e. larger incidence rates for the urban area and, within the rural area variation according to the degree of urbanization. There are, at present, very few data in the literature on affective psychoses and results are not consistent pointing to the need for more research in this area.

## Competing interests

The authors declare that they have no competing interests.

## Authors’ contributions

AS, JV, JRR, ML, PML and FS participated to the conception and design of the study, AS, ML and PML coordinated the study, TC, AMG, JV and JRR participated in the acquisition of data, AS performed the analyses and wrote the first draft of the manuscript. All authors participated in the writing and revision of the successive drafts of the manuscript and approved the final version.

## Pre-publication history

The pre-publication history for this paper can be accessed here:

http://www.biomedcentral.com/1471-244X/14/78/prepub
